# Rheological and Physicochemical Properties of Hyaluronic Acid Fillers for Body Contouring: Clinical Implications and Anatomical Considerations

**DOI:** 10.1111/jocd.70553

**Published:** 2026-02-19

**Authors:** Silvia Fontenete, Michael Alfertshofer

**Affiliations:** ^1^ Medical Department BioScience GmbH Madrid Spain; ^2^ Department of Plastic and Hand Surgery Technical University Munich Munich Germany

**Keywords:** body contouring, hyaluronic acid fillers, non‐surgical aesthetic procedures, rheological properties

## Abstract

**Introduction:**

The use of hyaluronic acid (HA) fillers is rising globally. Traditionally, the rheology of HA fillers has been subject to investigations for their use in facial soft tissues. Hitherto, there has been a significant gap in understanding their rheologic properties in body applications. The requirements for body fillers are different from facial fillers as they are applied for larger volume deficits, and experience greater mechanical stress. This study aims to fill this gap by analyzing the physicochemical and rheological properties of HA body fillers to guide clinical practice.

**Materials and Methods:**

Four commercially available HA‐based body fillers were analyzed under standardized laboratory conditions. The physicochemical properties, including pH, osmolality, ion concentrations, clarity, and swelling factor, were assessed. Rheological properties, including storage modulus (*G*′), loss modulus (*G*″), complex modulus (G*), and tan delta (tan δ), were assessed between 0.1 and 1 Hz.

**Results:**

The physicochemical analysis revealed no significant differences among the fillers, indicating uniform chemical stability. However, rheological analysis showed significant variations. Infini B Body exhibited the lowest *G*′, *G*″, and G* values. In contrast, HYAcorp MLF1 and MLF2 displayed higher *G*′ and G* values, indicating greater elasticity and stiffness. Consistent rankings between 0.1 and 1 Hz suggest stable mechanical performance under dynamic and sustained loading, supporting their suitability for high‐load applications such as gluteal augmentation.

**Conclusion:**

Selecting HA body fillers based on their rheological properties is crucial for optimizing outcomes, particularly in body contouring procedures where mechanical demands differ from those of facial applications. Clinicians should tailor filler selection to the specific requirements of each body area. Further research is required to validate these findings in long‐term clinical settings.

## 1 Introduction

The annual statistical report from the International Society of Plastic Surgeons (ISAPS) revealed that hyaluronic acid (HA) fillers account for 29.0% of all nonsurgical procedures worldwide with a 29% increase compared to the previous year. This trend has been consistently observed over the past five years, with HA injections showing a 28.9% overall increase during this period [1]. Although specific statistics on the use of HA fillers for body procedures remain limited, recent years have seen a notable increase in scientific literature addressing their application in body contouring. This growing interest highlights the expanding role of nonsurgical solutions for body contouring within the medical and aesthetic communities. The rise in studies mirrors the increasing number of patients opting for nonsurgical body procedures, driven by the advantages these treatments offer over surgical alternatives, including minimal downtime, immediate results, and reduced discomfort both during and after the procedure [2, 3, 4, 5, 6].

The rheology of HA has been studied since the 1960s [7]. Understanding physicochemical and rheological properties is essential, given their significant impact on the outcomes of aesthetic procedures [8, 9, 10, 11, 12]. Fillers impact tissue mechanics by altering the physical properties of the treated area through volume replacement, structural support, and integration with native tissues. The effects depend on the filler's rheological properties. HA fillers have distinct biophysical properties which have been summarized under measurable metrics such as elasticity modulus (*G*′), viscosity modulus (*G*″), and complex modulus (G*), which help the practitioner select the appropriate filler and injection method depending on the indication. *G*′ measures a filler's elasticity (its ability to store energy), *G*″ reflects its viscosity (its tendency to dissipate energy), and G* represents the overall stiffness by combining both elastic and viscous properties. Fully grasping these properties is essential for determining the filler's strength, firmness, and elasticity, which in turn affects its behavior in terms of tissue integration, longevity, and overall clinical results [13]. For example, in the face and body, HA fillers are subjected to low‐frequency forces, such as those caused by gradual muscle movements, tissue pressure, or gravity. A filler with a higher *G*′ offers better resistance to deformation and greater lifting capacity, making it suitable for structure and volume, whereas a filler with a higher *G*″ may provide better adaptability to surrounding tissues [13, 14].

While extensive research has been conducted on HA fillers for facial applications [10, 11, 15, 16], there is a noticeable lacuna in the literature regarding body‐specific HA fillers, which have different requirements for achieving optimal results. The significant differences between body‐specific and facial HA fillers underscore their tailored adaptation to the distinct anatomical and functional requirements of these regions. Facial anatomy, with its delicate skin, intricate musculature, and dense vascular network, necessitates fillers that integrate seamlessly with dynamic tissues and accommodate fine movements. In contrast, the thicker skin and substantial subcutaneous fat layers of body areas like the buttocks and thighs require fillers with enhanced structural support and lifting capacity. These anatomical distinctions are also mirrored in their respective injection techniques, with facial applications demanding precise, low‐volume injections and body contouring requiring higher volumes and often deeper tissue placement [4, 5]. Recognizing these differences is critical for selecting appropriate fillers and optimizing treatment outcomes in both facial and body applications. Moreover, these differences impose a unique set of rheological and physicochemical properties, such as higher viscosity and elasticity to maintain shape and resist deformation under pressure [13, 17]. However, the lack of comprehensive studies on these specific fillers means that practitioners may not have the detailed information needed to make fully informed decisions about product selection and application techniques, potentially impacting the effectiveness and safety of body contouring procedure [18].

This study aims to fill this gap by providing a comparative analysis of the physicochemical and rheological properties of four body HA fillers currently available on the market, ultimately guiding practitioners in selecting the most appropriate products for body contouring procedures.

## 2 Material and Methods

### 2.1 Study Design

A total of *n* = 4 injectable HA body fillers were analyzed in an independent laboratory under standardized conditions. The fillers were specifically chosen based on their reported characteristics by the manufacturers and are representative of leading brands with significant market share. The analysis included two monophasic (DeneB Classic‐H, BioPlus Co., Lt, Seoul, South Korea and Infini B Body, Infini, Milano, Lombardy, Italy) and two biphasic fillers (HYAcorp MLF1 and HYAcorp MLF2, BioScience GmbH, Dümmer, Lower Saxony, Germany), all with identical HA concentrations (Table 1). DeneB Classic‐H Filler is not an HA body filler, as its approved indication is limited to facial applications. However, since it is used off‐label for body treatments in various countries, we have included it in this study. To ensure accuracy, laboratory conditions were rigorously controlled, with the temperature consistently maintained at 21.0°C ± 1.5°C and relative humidity at 24.0% ± 5.0%.

**TABLE 1:** Overview of included materials.

Brand	Product commercial name	Cross‐linked HA concentration (mg/mL)	Non‐cross‐linked HA concentration (mg/mL)	Lidocaine (mg/mL)	Recommended area of use
BioPlus Co., Lt	DeneB Classic‐H	20	NA	NA	Facial tissue augmentation
Infini	Infini B Body	20	NA	NA	Body shaping and contouring, buttocks and calves
BioScience GmbH	HYAcorp MLF1	20	2	NA	Hip dips, calves, concave deformities
	HYAcorp MLF2	20	2	NA	Gluteus area, calves, concave deformities

Abbreviations: HA, hyaluronic acid; NA, not applicable.

The following materials were utilized: Dulbecco's phosphate‐buffered saline (PBS) without calcium and magnesium (Lonza Group, Basel, Switzerland), distilled water (Walter Schmidt Chemie GmbH, Berlin, Germany), 10 mL centrifuge tubes (Carl Roth, Karlsruhe, Germany), 1 mL syringes (Becton Dickinson, Franklin Lakes, New Jersey, USA), a connector (B. Braun Melsungen AG, Melsungen, Germany), and 26 G needles (TSK Laboratory, Tochigi, Japan).

### 2.2 Physicochemical Properties

The pH of each sample was measured in duplicate using a 913 Metronome pH meter. The swelling factor (SwF) was assessed by adding 1.5 mL of 1× PBS to the filler sample, followed by incubation at room temperature (23°C; 77% relative humidity) for 24 h. Post‐incubation, the PBS was removed, and the volume of the residual filler was measured with an automated electronic pipette. Clarity was evaluated in duplicate using narrow‐angle scattering with a BYK‐Gardner haze‐gard. The filler sample was positioned in front of the illumination unit, and measurements were taken using sensors located on the sphere side within the light trap area to capture narrow‐angle scattering behavior. Sodium (Na^+^) ion concentration was determined using an Agilent 5800 ICP‐OES system with appropriate calibration standards. Osmolality was measured in duplicate using a standard osmometer (model 2020; Advanced Instruments Inc., Norwood, MA).

### 2.3 Rheological Properties

The rheological properties of the body HA fillers were assessed using a Discovery Hybrid Rheometer (DHR) equipped with a controlled stress single head. A 60 mm parallel plate setup was utilized in conjunction with a Peltier plate made of aluminum, maintained at a constant temperature of 25°C. The tests were conducted at a strain of 2%, between 0.1 and 1 Hz (sampling 5 points per decade) to determine the *G*′, *G*″, and loss tangent (tan δ). A frequency of 1 Hz was included as it represents the standard reference condition used in rheological testing for facial indications, enabling comparability with existing literature. In contrast, 0.1 Hz was chosen to approximate the slower, sustained mechanical stresses and movements characteristic of the gluteal region during daily activities. The G* was calculated using the formula:

$$G^{*}=\sqrt{{\left(G^{\prime }\right)}^{2}+{\left(G^{\prime \prime }\right)}^{2}}$$

Method validation involved calculating the mean coefficient of variation (CV%) for four control samples in independent measurements conducted on the same date as the study samples.

### 2.4 Data Analysis

All experiments were performed in duplicate and analyzed using the R statistical package (R Foundation for Statistical Computing, Vienna, Austria) and GraphPad Prism (GraphPad Software Inc., California, USA). Arithmetic mean values for each product were calculated and are presented as ranges (physicochemical properties) or mean ± standard deviation (rheological properties). Normality was evaluated using the Shapiro–Wilk test, and descriptive analyses were conducted to summarize the data. The Kruskal–Wallis test, a nonparametric alternative to ANOVA, was employed for statistical comparisons between HA body fillers. Dunn's multiple comparison test was subsequently applied for pairwise comparisons.

## 3 Results

### 3.1 Physicochemical Properties

**FIGURE 1:**
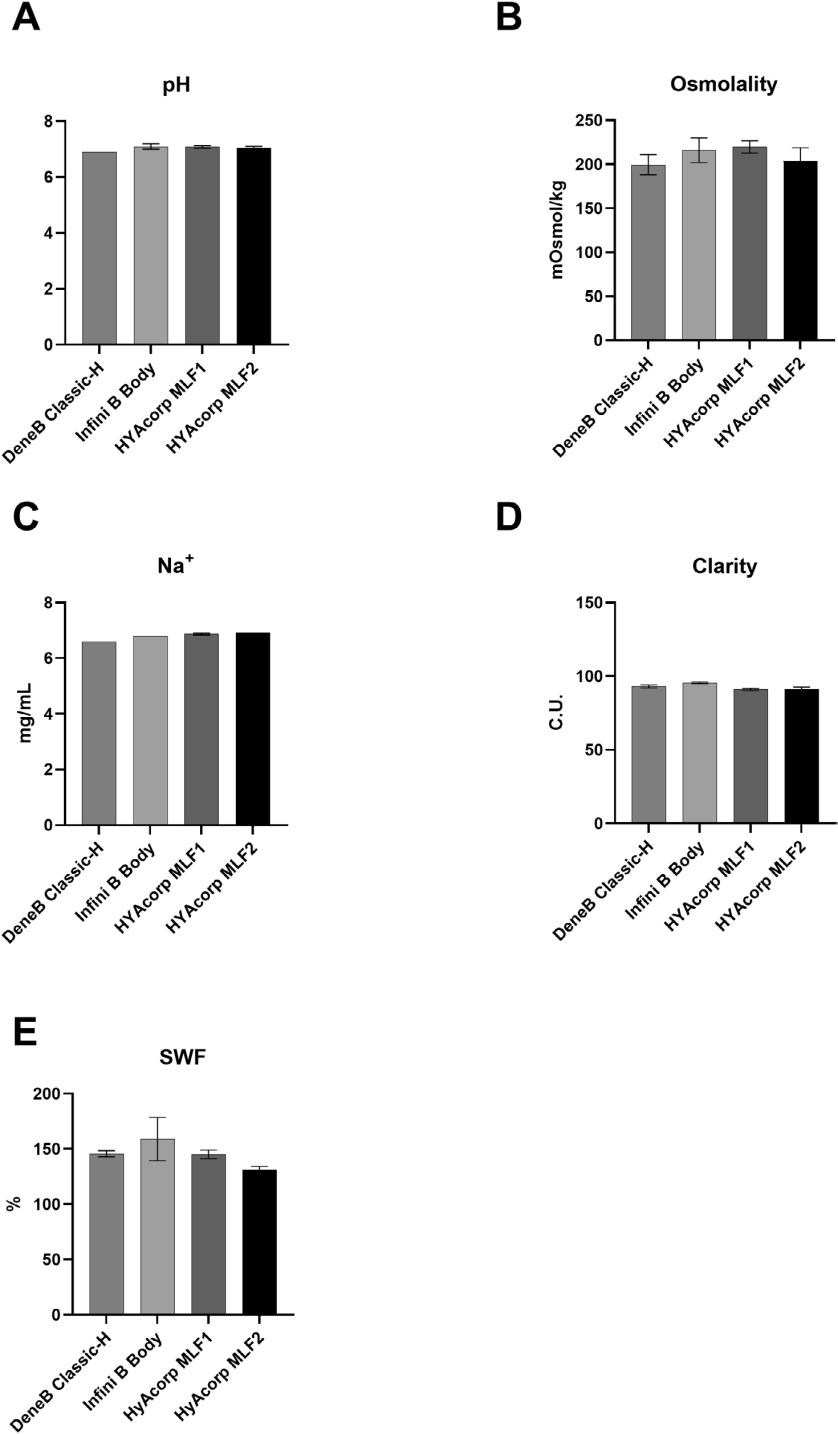
Physicochemical properties of the body HA fillers in the study. (A) pH; (B) Osmolality. (C) Sodium ions (Na^+^). (D) Clarity. (E) Swelling factor (SwF).

No statistically significant differences across all tested parameters, including pH, osmolality, Na^+^ ion concentrations, clarity, and SwF were found. The pH levels of all fillers were consistently within a narrow range of 6.9–7.2 (Figure 1A). Osmolality measurements exhibited slight variations, ranging from 188 to 233 mOsmol/kg (Figure 1B). Similarly, the concentrations of sodium ions were consistent and exhibited slight variations, ranging from 6.6 to 6.9 mg/mL (Figure 1C). Clarity assessments revealed minor differences but demonstrated overall similar optical transparency among the fillers, ranging from 89 to 96 (Figure 1D). Similarly, SwF measurements showed consistent results across the tested fillers, ranging from 136.4% to 178.6% (Figure 1E). These findings suggest a high degree of similarity in the physicochemical properties of the HA fillers analyzed, as detailed in Figure 1.

### 3.2 Rheological Properties

The rheological properties of the body filler in this study were analyzed between 0.1 and 1 Hz.

**FIGURE 2:**
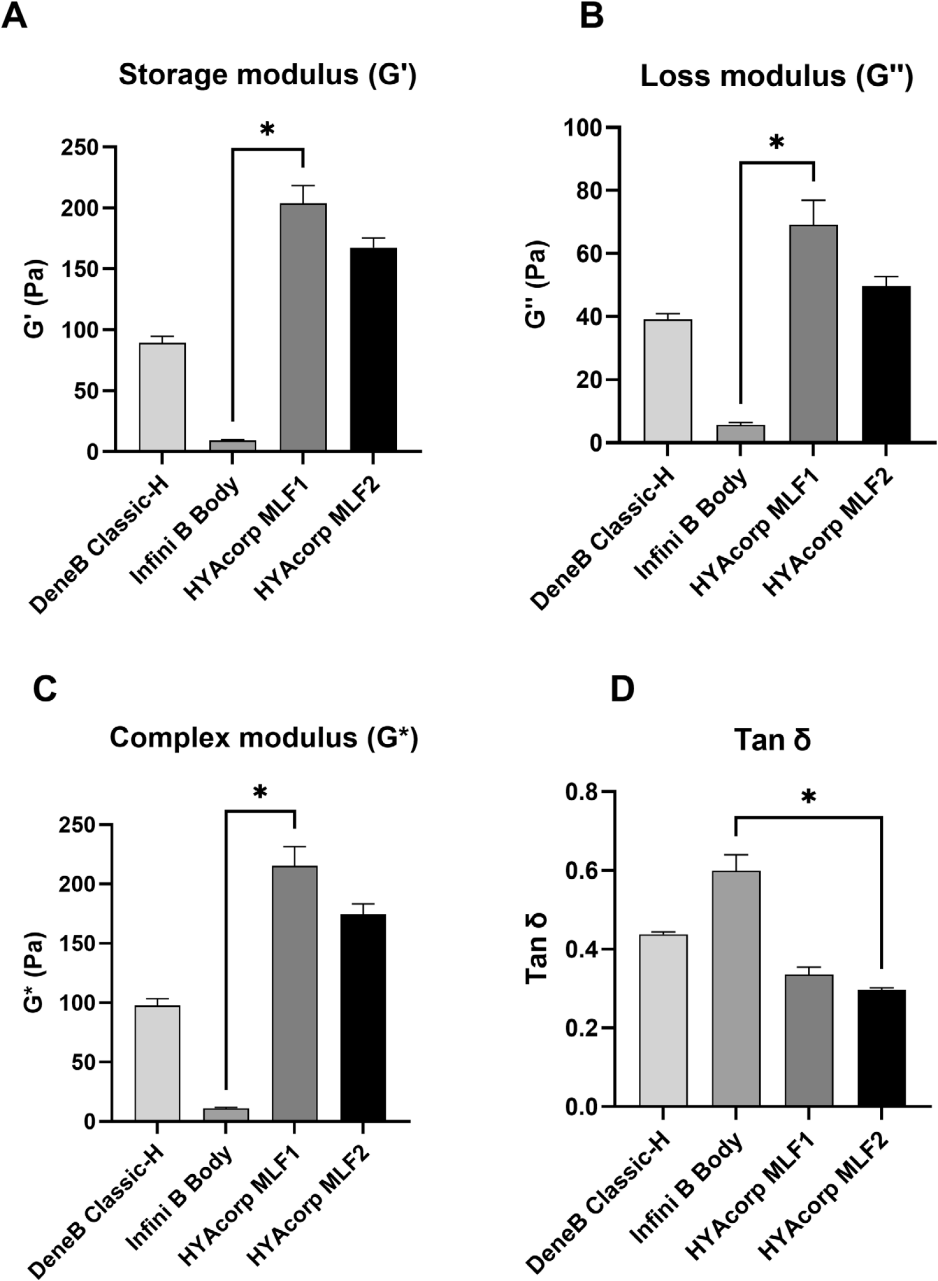
Comparison of the rheological characteristics of each product at 1 Hz. (A) Elastic storage modulus (*G*′); (B) Viscous loss modulus (*G*″). (C) Complex viscosity modulus (G*). (D) Tan δ. *p < 0.05.

The mean *G*′ values at 1 Hz demonstrated variations in the stiffness and structural support among the tested fillers. MLF1 exhibited the highest *G*′ (203.84 ± 35.80 Pa), followed by MLF2 (167.32 ± 20.24 Pa), DeneB Classic‐H (89.49 ± 5.41 Pa), and Infini B Body (9.28 ± 0.72 Pa). Infini B Body exhibited significantly lower *G*′ values compared to MLF1 (*p* = 0.028) (Figure 2A). In contrast, MLF1 showed the highest *G*′ values, though the difference between MLF1 and MLF2 or DeneB Classic‐H was not statistically significant (*p* = 0.874 and *p* = 0.124, respectively) (Figure 2A). The mean *G*″ values at 1 Hz revealed differences in the energy dissipation capacities among the tested fillers. MLF1 exhibited the highest *G*″ (69.12 ± 19.11 Pa), followed by MLF2 (49.74 ± 7.35 Pa), DeneB Classic‐H (39.13 ± 2.61 Pa), and Infini B Body (5.59 ± 1.15 Pa). Similarly, Infini B Body had significantly lower *G*″ values compared to MLF1 (*p* = 0.028) (Figure 2B). No other differences were observed (*p* > 0.05) (Figure 2B). The mean G* values at 1 Hz revealed significant differences in the overall viscoelasticity among the fillers. MLF1 exhibited the highest G* (mean: 215.40 ± 39.53 Pa), followed by MLF2 (174.57 ± 21.42 Pa), DeneB Classic‐H (97.67 ± 8.05 Pa), and Infini B Body (10.84 ± 1.46 Pa). Infini B Body's G* values were also significantly lower than those of MLF1 (*p* = 0.028), (Figure 2C). The difference between MLF1, MLF2 and DeneB Classic‐H was not statistically significant (*p* > 0.05). The mean tan δ values at 1 Hz highlighted differences in the balance between viscous and elastic properties among the fillers. Infini B Body demonstrated the highest tan δ (0.599 ± 0.058), followed by DeneB Classic‐H (0.438 ± 0.008), MLF1 (0.336 ± 0.046), and MLF2 (0.297 ± 0.013). Infini B Body demonstrated significantly higher tan δ values compared to MLF2 (*p* = 0.019). No significant differences were found between MLF1, MLF2 and DeneB Classic‐H (*p* > 0.05) (Figure 2D).

At 0.1 Hz, the rheological profile of the tested fillers showed trends similar to those observed at 1 Hz, with only minor variations in absolute values (Figure S1). MLF1 and MLF2 maintained the highest *G*′ values (141.70 ± 25.28 Pa and 121.00 ± 14.07 Pa, respectively), followed by DeneB Classic‐H (53.87 ± 4.89 Pa) and Infini B Body (4.61 ± 0.24 Pa). For *G*″, MLF1 (50.33 ± 11.85 Pa) and MLF2 (37.88 ± 5.64 Pa) again ranked highest, with DeneB Classic‐H (22.65 ± 2.06 Pa) and Infini B Body (2.38 ± 0.40 Pa) showing lower values. The G* values followed the same order, with at 150.40 ± 27.58 Pa, MLF2 at 126.80 ± 15.68 Pa, DeneB Classic‐H at 58.44 ± 5.31 Pa, and Infini B Body at 5.19 ± 0.39 Pa. Tan δ values were slightly higher for Infini B Body (0.515 ± 0.060) compared to the other fillers (*p* < 0.0001), which remained below 0.42. Overall, the relative ranking of products was consistent between 0.1 and 1 Hz (Figures 2, S1 and Table 2), suggesting stable viscoelastic performance across different frequency conditions relevant to both dynamic and sustained loading.

**TABLE 2:** *G*′, *G*″, G* and tan δ values (in Pascals) for the 4 measured body fillers at 0.16–0.6 Hz.

Product code	HYAcorp MLF1				HYAcorp MLF2				DeneB Classic H				Infini B Body			
Frequencies (Hz)	0.16	0.2	0.4	0.6	0.16	0.2	0.4	0.6	0.16	0.2	0.4	0.6	0.16	0.2	0.4	0.6
*G*′ (Pa)	151.8	162.8	175.2	189.0	128.8	138.9	146.6	156.8	59.3	65.5	72.5	80.5	5.23	6.0	6.9	8.0
*G*″ (Pa)	53.8	57.2	60.7	64.3	39.9	42.1	43.7	46.2	25.2	27.9	31.2	34.8	2.8	3.3	3.9	4.6
G* (Pa)	161.1	172.6	185.6	199.8	130.5	145.2	153.0	163.4	64.4	71.2	78.9	87.7	5.9	6.9	7.9	9.2
Tan δ	0.3	0.3	0.3	0.3	0.3	0.3	0.3	0.3	0.4	0.4	0.4	0.4	0.5	0.5	0.6	0.6

*Note:* Values represent mean values for measurements.

Abbreviation: Pa, Pascals.

## 4 Discussion

This study provides a comprehensive analysis of the physicochemical and rheological properties of four commercially available HA fillers specifically designed for body contouring, an area that has seen significant growth in demand in recent years [19]. DeneB Classic‐H Filler is not classified as an HA body filler, as its approved indication is limited to facial applications under its current regulatory authorization. However, because the product is widely used off‐label for body contouring procedures in several countries, including gluteal and hip augmentation, we included it in this study to better reflect real‐world clinical practice and provide a more comprehensive comparison across products frequently used in these indications. The present findings are crucial as they fill a gap in the existing literature, where research focused mainly on facial HA fillers, leaving the distinct requirements of body‐specific fillers underexplored.

The uniformity observed in the physicochemical properties across all tested HA body fillers suggests that these products have been optimized to meet general standards in the field. The consistency in pH, osmolality, ion concentrations, clarity, and swelling factor across the fillers indicates that these products (although with different compositions) are comparable in terms of their basic chemical stability and hence probably also biocompatibility. The rheological analysis, however, revealed significant differences among the fillers, particularly in their elastic and viscous properties, which are critical for their performance in body contouring procedures [20].

At 1 Hz, Infini B Body consistently showed lower *G*′, *G*″, and G* values compared to MLF1, suggesting that it is less elastic and more fluid‐like under mechanical stress [21]. This characteristic may limit its ability to maintain shape and volume, potentially making it less suitable for areas requiring high structural stability [21, 22, 23]. Conversely, MLF1 and MLF2 demonstrated the highest *G*′ and G* values, indicating superior elastic and overall stiffness properties [16]. These features might suggest that MLF1 and MLF2 may be better suited for applications requiring greater support and durability, such as in regions subjected to higher mechanical stress [11]. The differences observed between MLF1 and MLF2, although not statistically significant, point to subtle variations that could influence product selection depending on the specific clinical requirements. However, this difference does not necessarily contradict the clinical performance of MLF2 as the “higher‐lift” product. Lift capacity is inherently multifactorial, determined not only by elastic modulus but also by viscosity, cohesivity, and particle size distribution. MLF2 has a larger particle size range (300–500 μm) compared to MLF1 (200–350 μm), which can enhance volumizing capacity and tissue projection despite a lower *G*′. Moreover, parameters such as viscosity, cohesivity, and injection depth further influence the perceived firmness and lifting effect in vivo. DeneB Classic‐H, with its moderate *G*′ and G* (Figure 1A,C), provides a versatile alternative for cases requiring a balance between rigidity and adaptability, lying between the very soft profile of Infini B Body and the more rigid profiles of MLF1 and MLF2 fillers. It might be a reliable choice for subtle volume enhancement or moderate shaping. The tan δ values provided additional insights into the balance between elasticity and viscosity [13]. The higher tan δ values in Infini B Body indicate a greater viscous component, aligning with its more fluid‐like behavior [21]. This could be advantageous in applications requiring smoother integration with surrounding tissues but may be a drawback in scenarios where structural support is paramount. DeneB Classic‐H showed moderate tan δ suggesting it is well‐suited for applications requiring a balance of support and adaptability. This makes it an excellent choice for contouring where rigidity is not the primary requirement. MLF1 and MLF2, with their lower tan δ, emphasize elastic dominance, meaning the filler resists deformation and recovers its shape after stress, which is ideal for providing structural support and lift. Considering all the rheological properties, MLF1 and MLF2 fillers exhibit high *G*′ and G* values, indicating superior stiffness and elasticity. These characteristics make them suitable for regions subjected to significant mechanical stress, such as the buttocks or thighs, where shape retention and durability are critical. Conversely, Infini B Body, with lower *G*′ and higher tan δ values, demonstrates a softer and more adaptable profile, although its increased fluidity may reduce stability in areas of high motion [11]. DeneB Classic‐H exhibits a balanced rheological profile, combining stiffness and flexibility. This makes it suitable for moderate contouring applications within its regulatory approval, which is restricted exclusively to facial use.

The additional analysis between 0.1 Hz and 0.6—representing slower, sustained loading conditions—revealed the same relative ranking of fillers as at 1 Hz, with only minor variations in absolute values. MLF1 and MLF2 maintained the highest *G*′ and G* values, followed by DeneB Classic‐H and Infini B Body (Figure S1 and Table 2). This consistency across frequencies indicates that the mechanical behavior of the tested fillers is stable under both dynamic and low‐frequency conditions, supporting their suitability for clinical scenarios such as gluteal augmentation, where fillers are subjected to a combination of sustained gravitational loading and intermittent mechanical stress.

The findings from this study have several important clinical implications, particularly in the context of body contouring procedures, where the choice of filler can significantly influence the outcome [13]. The clinical effectiveness of a filler depends on its ability to withstand gradual forces without excessive flow (low *G*″) while maintaining adequate firmness and structure (high *G*′). Therefore, understanding the viscoelastic properties through clinically relevant rheological testing conditions is essential to optimize filler selection and ensure favorable outcomes in terms of tissue integration, longevity, and overall aesthetic results. Body HA fillers, unlike those used for facial treatments, are typically injected in larger volumes and need to endure more intense mechanical forces [2, 3, 4, 5, 6]. In high‐load, projection‐focused areas such as the gluteal region, rheological performance directly influences clinical longevity and aesthetic outcomes. In areas like the buttocks, fillers must sustain their shape and functionality despite the increased physical stress and movement [2, 3, 4, 5, 6]. This deeper placement requires fillers with specific rheological properties, such as increased crosslinking and a higher *G*′. Moreover, fillers with a high *G*′ and G* provide the stiffness and structural integrity needed to maintain projection under compressive forces, while an optimal tan δ balances shape retention with tissue integration. These properties are critical for sustaining contour and lift in dynamic conditions, supporting the selection of rheologically robust HA fillers for gluteal augmentation (Tables 3 and 4). Research supports that these enhanced properties are crucial for the fillers to remain stable and provide lasting volume in dynamic tissue environments like those found in body contouring areas such as the buttocks [4, 5, 6, 24]. The significant differences in rheological properties observed in this study, particularly in the *G*′, G*, suggest that not all HA fillers are equally suitable for areas requiring high structural support and long‐term volume retention.

**TABLE 3:** Key differences between facial and body HA fillers, highlighting their objectives, rheological properties, particle sizes, injection techniques, and anatomical suitability.

Characteristics	Facial fillers	Body fillers
Primary objective	Adaptability and natural integration	Structural support and volume retention
Elastic modulus (*G*′)	Balanced, lower to ensure flexibility	Higher for greater lifting capacity
Viscous modulus (*G*″)	Lower for smooth integration	Higher for durability and stability
Particle size	Small particle size	Larger particle size
Injection technique	Smaller volumes, superficial placement	Larger volumes, deeper placement
Anatomical considerations	Dynamic, delicate tissues (e.g., lips)	Thick skin, high‐stress areas (e.g., buttocks)

**TABLE 4:** Summary of key rheological parameters for hyaluronic acid‐based body fillers and their clinical relevance for high‐load, projection‐focused areas such as gluteal augmentation.

Rheological parameters	Clinical interpretation
Elastic modulus (*G*′)	Measure of filler elasticity (stiffness); higher *G*′ = greater shape retention, projection, and lifting capacity under pressure
Viscous modulus (*G*″)	Measure of filler viscosity (flow); higher *G*″ = better tissue adaptability and spreadability, but generally lower structural support
Complex modulus (G*)	Overall resistance to deformation (combination of elastic and viscous components); higher G* = greater firmness and stability under dynamic forces (e.g., sitting, movement)
Tan δ	Ratio of viscous to elastic behavior (*G*″/*G*′); low tan δ (< 1) indicates more elastic behavior, favoring shape maintenance, while high tan δ (> 1) indicates more viscous behavior, favoring integration and softness

The study also highlights the importance of tailoring filler choice to the specific needs and expectations of the patient. Clinicians should be aware of the differing properties of these fillers when planning body contouring procedures and should communicate these differences to patients. Furthermore, the rheological properties of the fillers could influence the long‐term outcomes and safety of the procedures [24]. Fillers that are too soft or fluid may migrate from the injection site over time, potentially leading to asymmetry or the need for corrective procedures [25]. Conversely, fillers that are too stiff may not integrate as smoothly with the surrounding tissues, potentially leading to palpable or visible irregularities [13].

The results of this study underscore significant differences in the rheological properties of body‐specific HA fillers compared to facial HA fillers, highlighting their adaptation to distinct anatomical and functional requirements. The anatomical and functional distinctions between facial and body regions necessitate the use of HA fillers with tailored rheological properties to achieve optimal clinical outcomes. Facial anatomy is characterized by intricate structures, including delicate skin, complex musculature, and a rich vascular network [26]. Facial fillers prioritize adaptability and natural integration, making them suitable for dynamic and delicate tissues, while body fillers are optimized for structural support and volume retention in areas with thicker skin, more substantial subcutaneous fat layers and higher mechanical stress. These distinctions highlight the importance of selecting the appropriate filler based on the treatment area and clinical requirements.

In facial applications, precise injection techniques are crucial to navigate the complex anatomy and to avoid complications [22]. For instance, subcutaneous injections are often employed to restore facial volume, but the abundant vasculature in the subcutaneous layer poses challenges, necessitating careful identification of arterial pathways. The selection of HA facial fillers is influenced by the need for products that can integrate seamlessly into the dynamic and delicate facial tissues. The rheological properties of these fillers are tailored to accommodate facial movements and to minimize the risk of complications. In body contouring, injections typically involve larger volumes and are administered into deeper tissue planes to achieve the desired augmentation. The thicker skin and more substantial subcutaneous tissues in body areas require fillers with different rheological profiles compared to those used on the face. HA‐based fillers for body contouring are engineered to withstand the unique mechanical stresses of the body, including slow and sustained deformation from gravitational forces and tissue compression. The rheological characteristics of these fillers are adjusted to meet the specific demands of body contouring procedures. Therefore, informed product choice coupled with a deep understanding of the interplay between rheology and tissue demands remains paramount for the treating physician to achieve the best possible aesthetic outcome while maintaining procedural safety.

This study provides valuable insights into the physicochemical and rheological properties of HA fillers for body contouring, addressing a significant gap in the literature where most research has focused on facial fillers. The findings offer critical guidance for clinical practice, particularly in selecting appropriate fillers based on their mechanical behavior under different conditions. However, the study's limited sample size, in vitro design, and focus on short‐term properties present limitations, suggesting the need for further research with more diverse products and long‐term in vivo analyses. Additionally, while the study highlights key differences among the fillers, it does not cover the full range of available products or directly correlate these properties with clinical outcomes, emphasizing the importance of continued research to fully understand and optimize the use of HA fillers in body contouring procedures. Moreover, although *G*′ and *G*″ characterize postinjection behavior, they do not serve as direct indicators of injectability. To better assess clinical handling, future research should include measurements of shear viscosity, extrusion force, and cohesive index [10, 27, 28]. Furthermore, the results should be interpreted with caution due to potential variability in manufacturing processes, cross‐linking technologies, and raw material sources among different brands, which may influence physicochemical and rheological properties. The in vitro conditions used in this study may not fully replicate the complex biological environment, including tissue integration, enzymatic degradation, and inflammatory responses, which can significantly impact filler performance over time. Differences in practitioner technique, injection depth, and patient‐specific anatomical factors were not addressed, and may alter clinical outcomes. While we included measurements at 1 Hz (the standard for facial filler rheology), we also assessed 0.1 Hz and intermediate low frequencies (0.2–0.5 Hz) to approximate slower, sustained loading in the gluteal region, as body contouring applications may additionally require evaluation under static load via creep testing to better reflect clinical conditions Lastly, the absence of standardized protocols for rheological and handling measurements across studies limits direct comparability with previously published data, underscoring the need for harmonized testing methodologies in future research. It is important to highlight that the use of HA fillers in non‐authorized indications carries inherent risks, as clinical safety and performance have not been established for those applications. In this article, we analyze only the rheological properties of the four HA fillers included, and no clinical conclusions should be drawn regarding their safety or efficacy in off‐label body indications.

## 5 Conclusion

This study underscores the importance of selecting HA fillers based on their rheological properties, particularly in body contouring procedures where the mechanical demands differ significantly from facial applications. The differences observed among the fillers studied suggest that a one‐size‐fits‐all approach is not appropriate. Instead, clinicians should consider the specific needs of each treatment area, the patient's aesthetic goals, and the long‐term implications of filler behavior. By doing so, they can optimize both the short‐term and long‐term outcomes of body contouring procedures, thereby enhancing patient satisfaction and safety.

## Author Contributions

S.F. designed the study and analyzed the results. S.F. and M.A. wrote the manuscript. All the authors reviewed and approved the final version of the manuscript.

## Conflicts of Interest

The authors declare no conflicts of interest.

## Supporting information


**Figure S1:** Comparison of the rheological characteristics of each product at 0.1 Hz. (A) Elastic storage modulus (*G*′); (B) Viscous loss modulus (*G*″). (C) Complex viscosity modulus (G*). (D) Tan δ. *p < 0.05.

## Data Availability

The data that support the findings of this study are available on request from the corresponding author. The data are not publicly available due to privacy or ethical restrictions.
