# Supplementary figures and images for: Rheological and Physicochemical Properties of Hyaluronic Acid Fillers for Body Contouring: Clinical Implications and Anatomical Considerations
Source: J Cosmet Dermatol. 2026 Feb 19;25(2):e70553. doi: 10.1111/jocd.70553 (PMC12921345; doi:10.1111/jocd.70553)

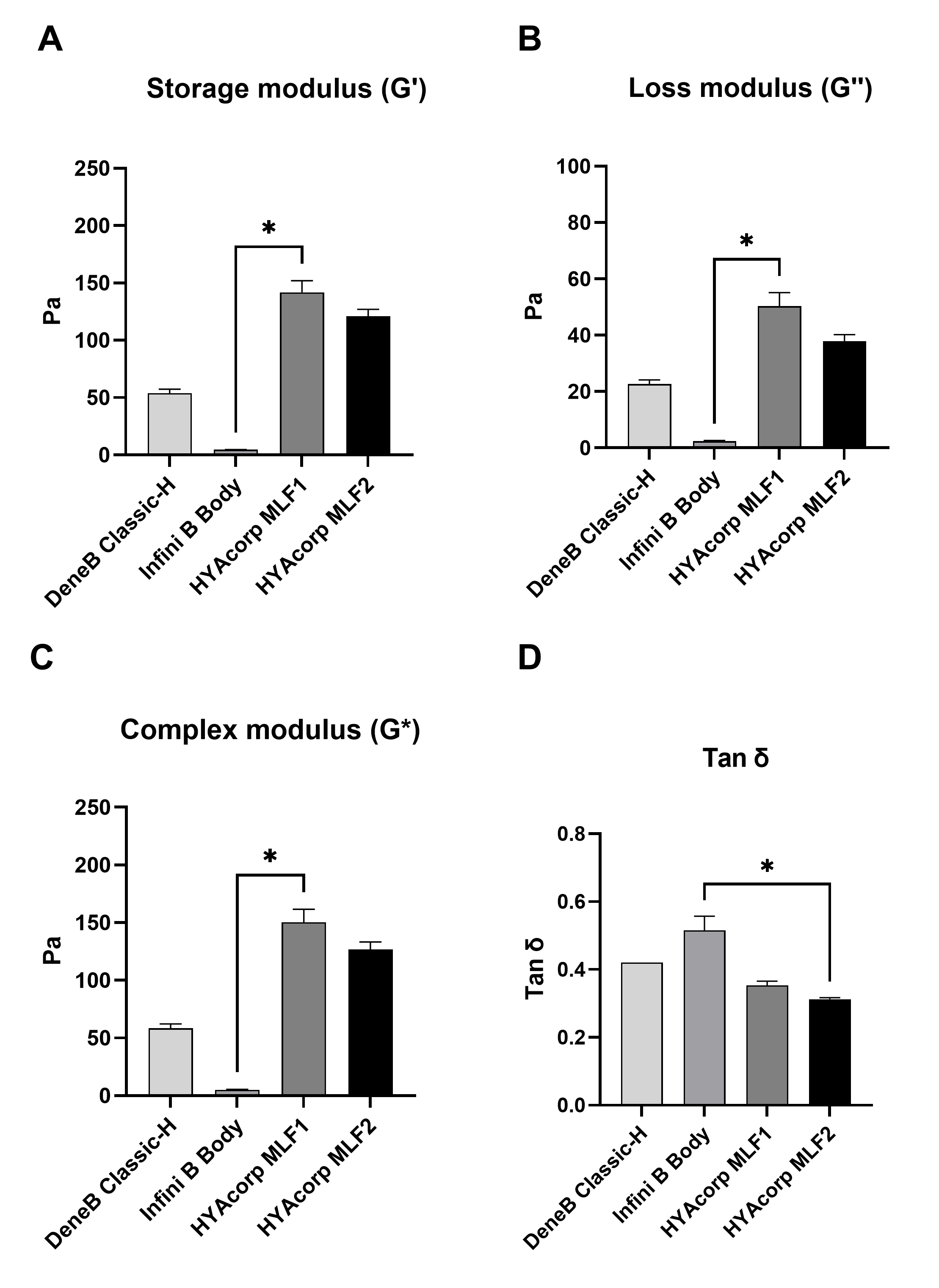

Supplement: Supplementary file 1 — Figure S1: Comparison of the rheological characteristics of each product at 0.1 Hz. (A) Elastic storage modulus (G′); (B) Viscous loss modulus (G″). (C) Complex viscosity modulus (G*). (D) Tan δ. *p < 0.05. [file JOCD-25-e70553-s001.tif]
